# Continuous Production of Bifunctional Platform Chemicals From Plant Oils in Water by Cyclodextrin‐Mediated Hydroformylation

**DOI:** 10.1002/cssc.202402421

**Published:** 2025-01-23

**Authors:** Thomas Friedrich Hubertus Roth, Tobias Averbeck, Marvin Daalmann, Dieter Vogt, Thomas Seidensticker

**Affiliations:** ^1^ TU Dortmund University Department for Biochemical and Chemical Engineering Laboratory of Industrial Chemistry Emil-Figge-Straße 66 44227 Dortmund Germany

**Keywords:** Homogeneous catalysis, Oleochemistry, Catalyst recycling, Miniplant, Design-of-experiments

## Abstract

Platform chemicals from renewable resources with broad applications are highly desirable, particularly for replacing fossil‐based monomers. Bifunctional aliphatic ester‐aldehydes, accessible *via* regioselective hydroformylation of unsaturated oleochemicals, can be converted into linear ω‐amino/ω‐hydroxy esters and dicarboxylic acids—key building blocks for biobased aliphatic polycondensates. However, their success hinges on efficient, economically viable production, with catalyst recycling being critical. We present the Rh‐catalyzed, cyclodextrin‐mediated, aqueous biphasic hydroformylation of methyl 10‐undecenoate (from castor oil) and methyl 9‐decenoate (from rapeseed oil) to produce methyl 12‐oxododecanoate and methyl 11‐oxoundecanoate, respectively, with high yields and productivity. This system allows for efficient catalyst recycling via decantation, maintaining 30 % of its native activity in aqueous biphasic conditions. Reaction conditions were optimized using a tailored experimental design, reducing nearly 200 experiments to 39 without sacrificing predictive accuracy. The optimized conditions were transferred to a continuous miniplant, achieving a low rhodium loss of 0.018 % h^−1^, with excellent space‐time yields of 76.5 kg h^−1^ m^−3^. Rhodium in the product was as low as 79 ppb, with 4.4 kg of product per mg of catalyst lost, marking a significant step in combining hydroformylation‐derived, bio‐based platform chemicals with economic industrial potential.

## Graphical abstract

Cyclodextrin‐mediated aqueous biphasic systems are a potent way to recycle homogeneous catalysts, also applicable to producing highly demanded renewable chemical intermediates. Based on a sophisticated batch optimization, a total STY of 76.5 kg/h/m^3^ was achieved in a continuously operated miniplant with minimal leaching of 0.018 %/h.

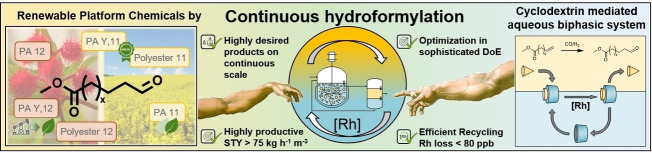

## Introduction

Renewable platform chemicals are key to establishing increasingly sustainable and efficient value chains for the future chemical industry. Commonly, biomass‐derived chemicals, such as 5‐hydroxymethylfurfural (5‐HMF) from hemicellulose, and products derived from it, such as levulinic acid, are discussed in this context.[^1^, ^2^, ^3^] However, for those chemicals to be implemented into the chemical value chain, substantially new processes must be developed, and entirely new products are generated, making it a long‐term strategy.^[3]^ On the other side, climate change and net‐zero regulations call for all chemists worldwide to take immediate action in developing fossil‐free alternatives to existing chemical production routes to make an impact now.[^4^, ^5^]

We are deeply convinced of the importance of bio‐based platform chemicals, such as those derived from hemicellulose, which have been widely discussed. However, given the pressing circumstances, we find it worthwhile to explore alternative platform chemicals that have so far received less attention but could offer a short‐term integration of renewable resources into the value chain. These alternatives are particularly valuable if they meet several key criteria: they must be available in reliable quality and quantity now or in the near future; at least some of the products derived from these platform chemicals should serve as drop‐in replacements for existing compounds, allowing for rapid implementation in current processes and fostering acceptance; and there should be a broad range of applications, including the potential for developing new products. In addition to these material considerations, the process for synthesizing these platform chemicals must also meet specific criteria for short‐term implementation: it must be scalable, reliable, sustainable, and robust, meaning it should be applicable to multiple platform chemicals. Furthermore, short development times in the laboratory are desirable for the process.

We envision that one possible approach to fulfilling all of these criteria lies in the development of a process for the hydroformylation of terminally unsaturated esters derived from vegetable oils. These oleochemicals are already available in large quantities and in the required quality. Additionally, the resulting bifunctional aldehyde‐esters can be converted into products such as amines, esters, and primary alcohols, allowing them to be used as monomers in novel polycondensates. Moreover, with appropriate chain lengths of the starting substrates, they can even serve as direct drop‐in alternatives to currently petrochemically produced monomers.

**Figure 1:**
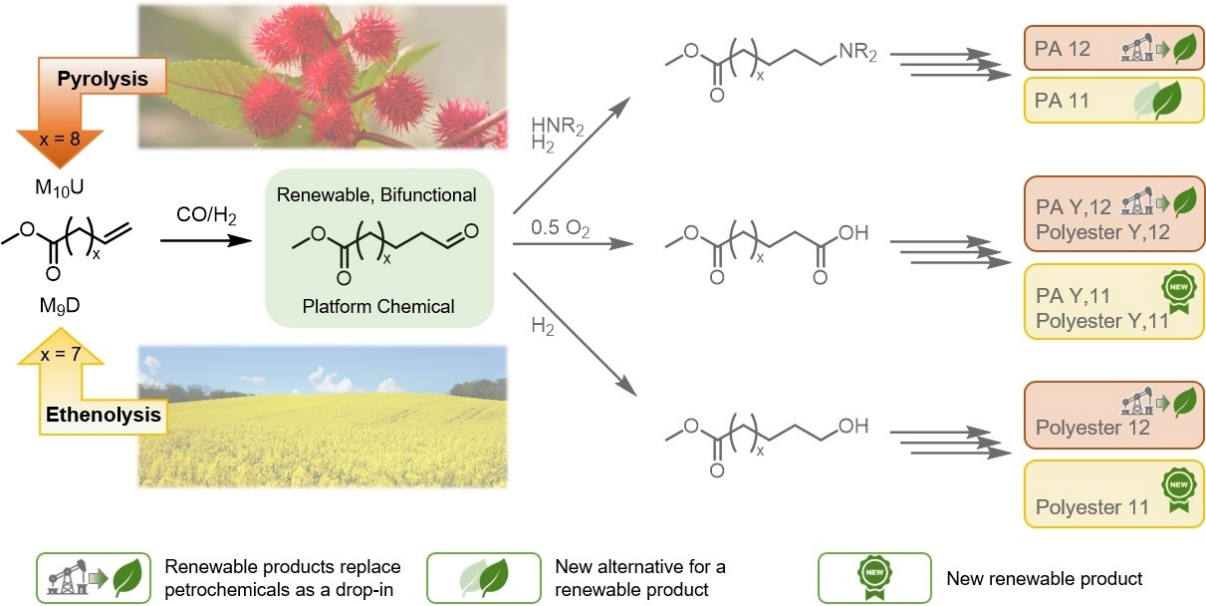
Linear, aliphatic ester‐aldehydes as renewable platform chemicals derived from the hydroformylation of unsaturated oleochemicals offer a broad field of application in different polycondensates.

Examples include terminally unsaturated methyl 10‐undecenoate (M_10_U), which is derived from castor oil and is currently used on an industrial scale only in polyamides (e. g., Rilsan®, Polyamide 11). Another oleochemical suited for this approach is obtained from the ethenolysis of locally sourced vegetable oils: methyl 9‐decenoate (M_9_D), which was recently introduced to the market by Germany's largest biodiesel producer, Verbio.^[6]^ Hydroformylation of these two substrates (Figure 1) would yield the bifunctional ester aldehydes methyl 12‐oxododecanoate (X=8) and methyl 11‐oxoundecanoate (X=7) as renewable platform chemicals for the synthesis of a range of bio‐based polycondensates.

Although the M_10_U and M_9_D hydroformylation products offer a promising and wide range of applications, no efficient processes exist to achieve high productivity simultaneously with an efficient recycling of the homogeneous transition metal catalyst.[^7^, ^8^] As mentioned, however, an efficient process plays a crucial role in the implementation of these platform chemicals to ensure both rapid and sustainable economic success. A key factor for success, particularly in hydroformylation, is the efficient recycling of the catalyst while maintaining consistently high productivity, long‐term stability, and robustness concerning the substrates used.^[9]^ A fundamental requirement is separating products and catalysts into two distinct phases post‐reaction. Various strategies have been investigated in general for the hydroformylation to achieve this separation, differentiated primarily by whether the phase designated for the catalyst is created after (e. g., thermomorphic multiphase system (TMS)[^10^, ^11^, ^12^, ^13^] precipitation,[^9^, ^14^, ^15^] distillation,[^9^, ^15^, ^16^] membrane processes[^9^, ^13^, ^17^]) or before (e. g., supported ionic liquid phase (SILP)[^18^, ^19^, ^20^] heterogeneously supported,[^21^, ^22^, ^23^] (aqueous) biphasic systems) the reaction.^[24]^ In the former case, reactions can be conducted efficiently in a single phase, with the main challenge being to generate two separate phases after the reaction. In the latter case, separation is usually straightforward due to the distinct phases of the catalyst and products. However, the reaction itself is often limited because of the challenging contact of the substrate and catalyst, which is why a variety of intensification strategies (intensified mixing[^25^, ^26^, ^27^] co‐solvents,[^26^, ^27^] phase transfer agents such as cyclodextrins[^28^, ^29^]) are being investigated, especially for (aqueous) biphasic systems.

**Figure 2:**
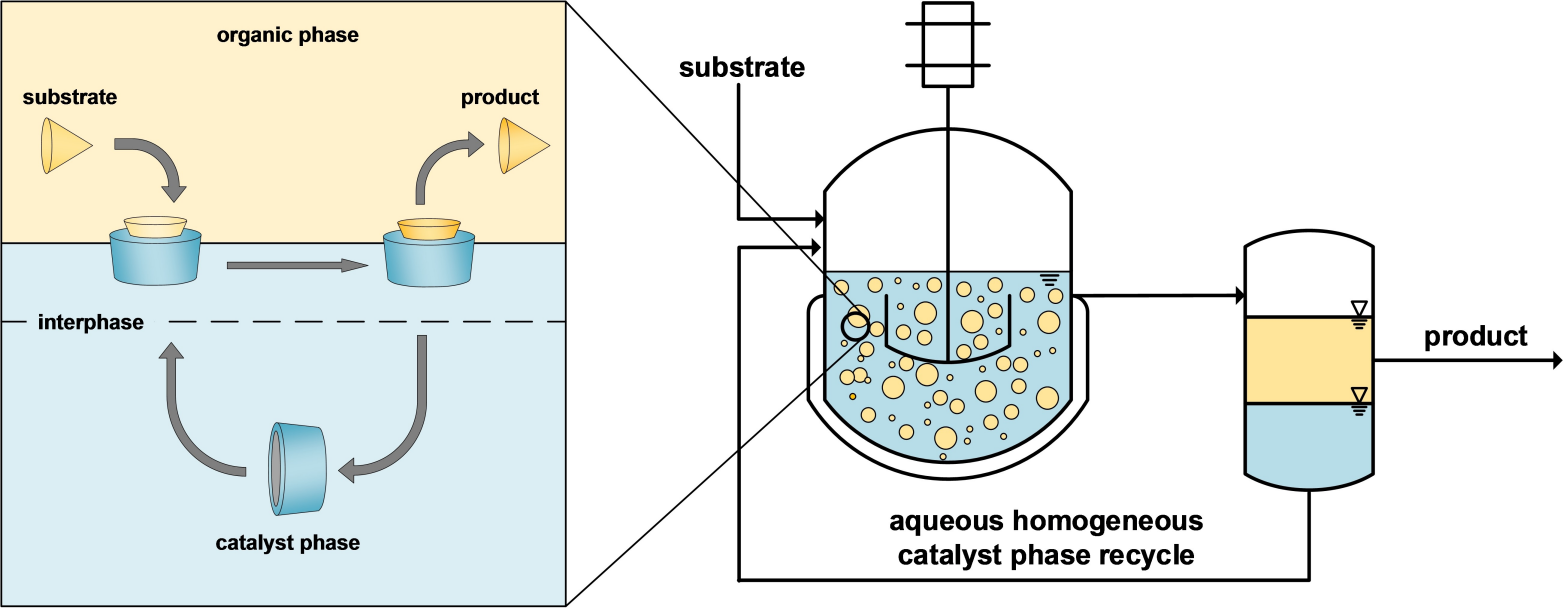
Postulated mechanism in the cyclodextrin‐mediated biphasic reaction system (left).[^29^, ^35^] A basic flow sheet for the recycling of homogeneous catalysts in aqueous biphasic reaction systems is used in this work (right).^[35]^

We have recently worked very successfully with randomly methylated *β‐*cyclodextrins as an intensification strategy, which achieved very low catalyst losses in continuous processes without compromising the overall catalytic performance.^[30]^ Here, macroscopically, two distinct phases are present during the reaction: (1) a non‐polar phase, containing mainly the substrate and products, and (2) an aqueous polar phase, containing the homogeneous catalyst and cyclodextrins (CD). These highly water‐soluble, cyclic glucose oligomers exhibit a conical cavity and thus can form an inclusion complex with non‐polar substrate molecules (Figure 2, left).[^29^, ^30^] This behavior significantly enhances the availability of non‐polar substrates in the polar catalyst phase while ensuring an excellent separation between the nonpolar products and the polar catalysts.[^31^, ^32^, ^33^] These systems have proven successful in continuous operation in our earlier published hydroformylation of 1–decene,^[34]^ with yields of about 40 % at 0.003 % h^−1^ leaching, resulting in a productivity of 1.5 kg of product per milligram of Rh loss to the product and in hydroaminomethylation (tandem reaction of hydroformylation and reductive amination), where an increase in the CD/Rh‐ratio from 15–250 reduced the time to complete conversion from 24 hours to less than 2 hours.^[35]^ Furthermore, no indications of leaching or degradation of CD have been observed in our studies. The expense for CD is therefore considered non‐recurring and benefits from the fact that they are widely used in other industries. It is therefore estimated that they are already available at bulk prices in the range of 110–140 € kg^−1^.

We believe that combining the establishment of bifunctional ester‐aldehydes as renewable platform chemicals for the synthesis of bio‐based polycondensates with their highly efficient production in a continuous, aqueous‐biphasic, cyclodextrin‐mediated process can significantly contribute to the defossilization of the chemical industry. This is the focus of our current study, and based on an optimized experimental design, we first determined optimal conditions in a batch DoE, which we then applied to continuous operating conditions.

## Results and Discussion

Based on our extensive experience in the field, we selected the well‐established Rh‐sulfoXantphos system for our study. This choice ensures two critical aspects: (1) high regioselectivity, with high yields of the highly desired linear aldehyde, and (2) reliable catalyst retention in the aqueous phase owing to the sulfonated nature of the catalyst. Potential side reactions include hydrogenation, isomerization, and alcohol formation.

### Parameter Optimization by Design‐of‐Experiments

**Figure 3:**
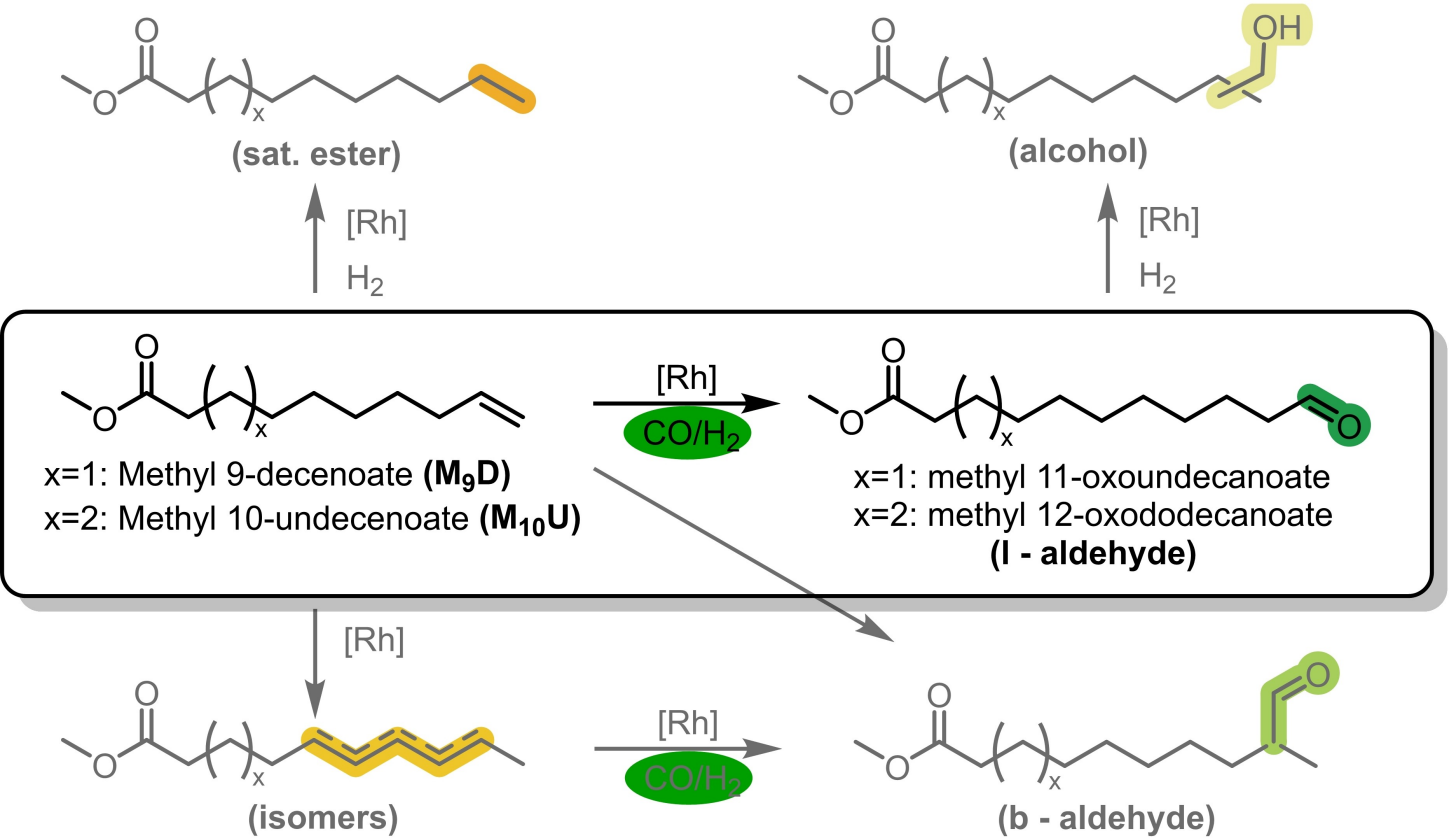
By hydroformylation of the renewable substrates M_9_D and M_10_U linear aldehydes are formed, which are valuable platform chemicals. For clarity, only one possible branched aldehyde is shown.

We demonstrated the reaction's fundamental feasibility in converting M_10_U in an initial batch experiment (Figure 3). However, the low activity of the reaction indicates an urgent need for optimization before the continuous operation. Therefore, we conducted a structured design of experiments (DoE), and drawing from our prior studies, we selected and varied several critical parameters to ensure that their ranges would likely encompass the optimum conditions. The key variable was the CD/Rh ratio, which was previously shown to impact the reaction rate significantly. We explored CD/Rh ratios between 100 and 750; higher values resulted in such viscous solutions that practical operation with our methodology was no longer feasible.

Temperature, influencing inclusion complex formation and reaction kinetics, was optimized within the 100–160 °C range. While lower temperatures are known to reduce activity, higher temperatures promote catalyst deactivation. The catalyst concentration was capped at 2.2 mmol/L, reflecting high activities observed in preliminary experiments, with lower concentrations also tested to enhance economic viability. The phase ratio (organic/aqueous) was investigated in a range of 0.2–0.8 due to its potential to increase productivity significantly. However, previous hydroaminomethylation (HAM) research indicated that high phase ratios could lead to excessive isomerization.^[36]^

While optimization targeting solely one key figure, e. g., selectivity, is well established in academic literature, a more sophisticated approach is needed to yield optimal conditions for continuous operation. Therefore, a more comprehensive optimization approach that simultaneously considers multiple key figures is essential for achieving optimal performance under continuous operating conditions. Multi‐objective optimization uses advanced algorithms that balance trade‐offs between competing objectives. Techniques such as Pareto optimization, genetic algorithms, and machine learning models can be used to explore the solution space and determine the most effective operating strategies.

To adequately address the complex interplay in the explored reaction system, we optimized not for a single variable but a composite objective function, including selectivity (S), STY, turnover number (TON), and leaching (L), which are combined in the target value Z_tot,i_ (see Supplementary Information for details). For the economic success of a process, the relative weighting of the individual factors would have to be determined by a comprehensive techno‐economic evaluation of all relevant factors; in the context of this work, this was done according to an empirical approach.

**Figure 4:**
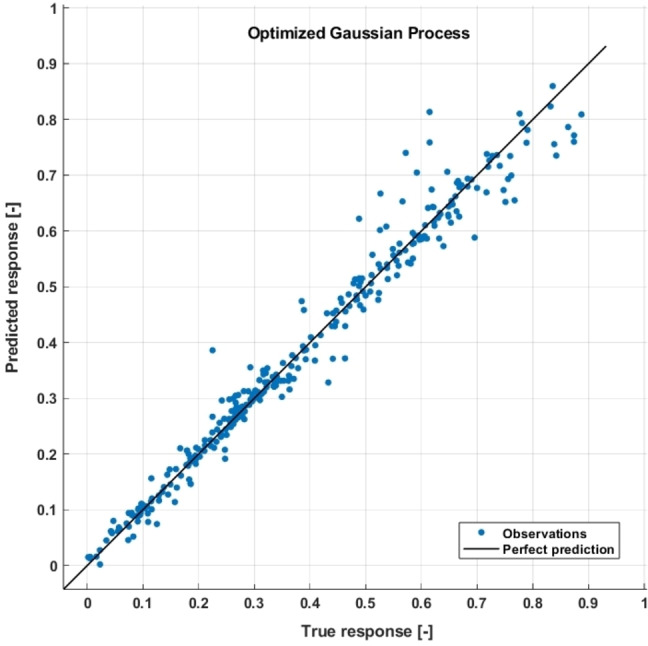
Parity plot of the results obtained from the model calculation *via* the Gaussian Process with the experimental data.

An extensive experimental plan initially required more than 200 individual experiments. However, using an algorithm for *d*‐optimal experimental design, we reduced this to 39 experiments while still ensuring high predictive reliability.[^37^, ^38^] A Gaussian Process (GP) was used to model the target variable Z since GPs are inherently well‐suited for modeling natural phenomena like chemical reactions. This is because they assume a continuous, smooth underlying process, which mirrors the behavior of many physical and chemical systems. The resulting GP model showed excellent representation of the experimental data, with a root‐mean‐square error (RMSE) of 0.037 and a coefficient of determination (R^2^) of 0.97 (Figure 4).

**Figure 5:**
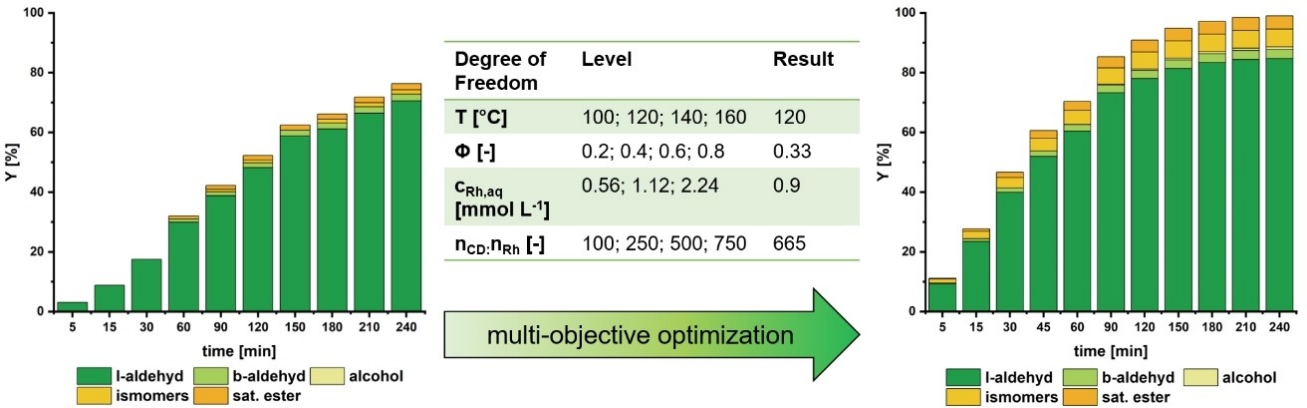
In the cyclodextrin‐mediated aqueous biphasic hydroformylation of M_10_U, productivity was significantly increased through focused multiobjective optimization compared to earlier published results in the hydroformylation of 1‐decene.^[34]^ Yields are calculated based on GC‐FID analysis of the product mixture with dibutyl ether as an internal standard. Conversion equals the sum of all yields. Conditions: left: T=120 °C, p=30 bar, u=800 min^−1^, n_CO_:n_H2_=1 : 1, c_Rh,aq_=2.2 mmol L^−1^, n_CD_:n_Rh_=12, ϕ=0.2, n_Sub_:n_Rh_=500, n_P_:n_Rh_=5; Preforming: T=120 °C, p=20 bar, u=800 min^−1^, t=1 h; right: T=120 °C, p=30 bar, u=800 min^−1^, n_CO_:n_H2_=1 : 1, c_Rh,aq_=0.9 mmol L^−1^, n_CD_:n_Rh_=665, ϕ=0.34, n_Sub_:n_Rh_=2500, n_P_:n_Rh_=7; Preforming: T=120 °C, p=20 bar, u=800 min^−1^, t=1 h.

A two‐stage optimization approach was employed, combining a genetic algorithm with a global search method to identify optimal operating parameters. This approach efficiently explored the parameter space and converged on robust, optimal conditions. The implementation details are available in the Supplementary Information to ensure reproducibility (see SI). Post‐optimization batch experiments demonstrated significant improvements, with the yield (Y) increasing from 70 % to 85 %, the TON increasing from 393–2,415, and the TOF_20_ increasing from 183 h^−1^ to 2,381 h^−1^ (Figure 5).

It is particularly noteworthy that by drastically increasing the cyclodextrin to rhodium ratio, resulting in a mass fraction of 1.88 g CD to 1 g of water, the absolute amount of catalyst could be more than halved while at the same time increasing the substrate loading, which drastically increased the overall TON and TOF_20_.

Under these optimized conditions, comparative results of 85 % yield and a TON of 2460 were achieved for the hydroformylation of M_9_D (see SI). Only the TOF_20_ of 1860 h^−1^ was slightly reduced in the batch experiment, whereas there were no significant differences in the catalyst leaching or the other reaction parameters compared to the hydroformylation of M_10_U, for which the conditions were optimized. When an equimolar mixture of M_9_D and 1‐decene, imitating an ethenolysis mixture of methyl oleate, is used, the same performance is observed for the former, while 75 % yield is also achieved for the latter after 240 min. Surprisingly, M_9_D achieves a significantly higher activity than 1‐decene in direct comparison (see SI). This could be due to a higher solubility of the methyl ester in the catalyst phase or different inclusion complex equilibria with cyclodextrins, which is currently the subject of ongoing investigations.

With this systematic optimization, 30 % of the native catalyst activity can now be achieved for the hydroformylation of M_10_U with the biphasic system. To determine this, we conducted an experiment under monophasic conditions in which the solvent water was replaced by toluene, the ligand sulfoXantphos was replaced by Xantphos and the addition of cyclodextrins was omitted (see SI). Based on this, it is assumed that the reaction is still limited by the substrate supply and not by the native catalyst activity. Nevertheless, this high activity is a very impressive result compared to the monophasic system, considering that the catalyst in the biphasic system can be recycled after simple decantation, whereas recycling in the monophasic system is highly demanding, if not practically impossible.

### Scale Up and Transfer to Continuous Miniplant Operation

For the migration to continuous operation in our miniplant plant (Figure 2, right, detailed description of the setup in our previous publication^[35]^), reliable phase separation within the residence time in the decanter and low catalyst loss are essential. Since both the viscosity of the catalyst phase and the partition coefficient of the catalyst have a considerable temperature dependence, the selected separation temperature is of crucial importance: Low temperature leads to low catalyst leaching; however, if the viscosity increases too much, the decanter may no longer be reliably operable. In contrast, higher temperatures result in reliable separation due to lower viscosity but potentially higher leaching. After preliminary investigations regarding the temperature‐dependent viscosity of the catalyst phase (see SI) and possible operating conditions in the miniplant, a separation temperature of 30 °C was finally set.

**Figure 6:**
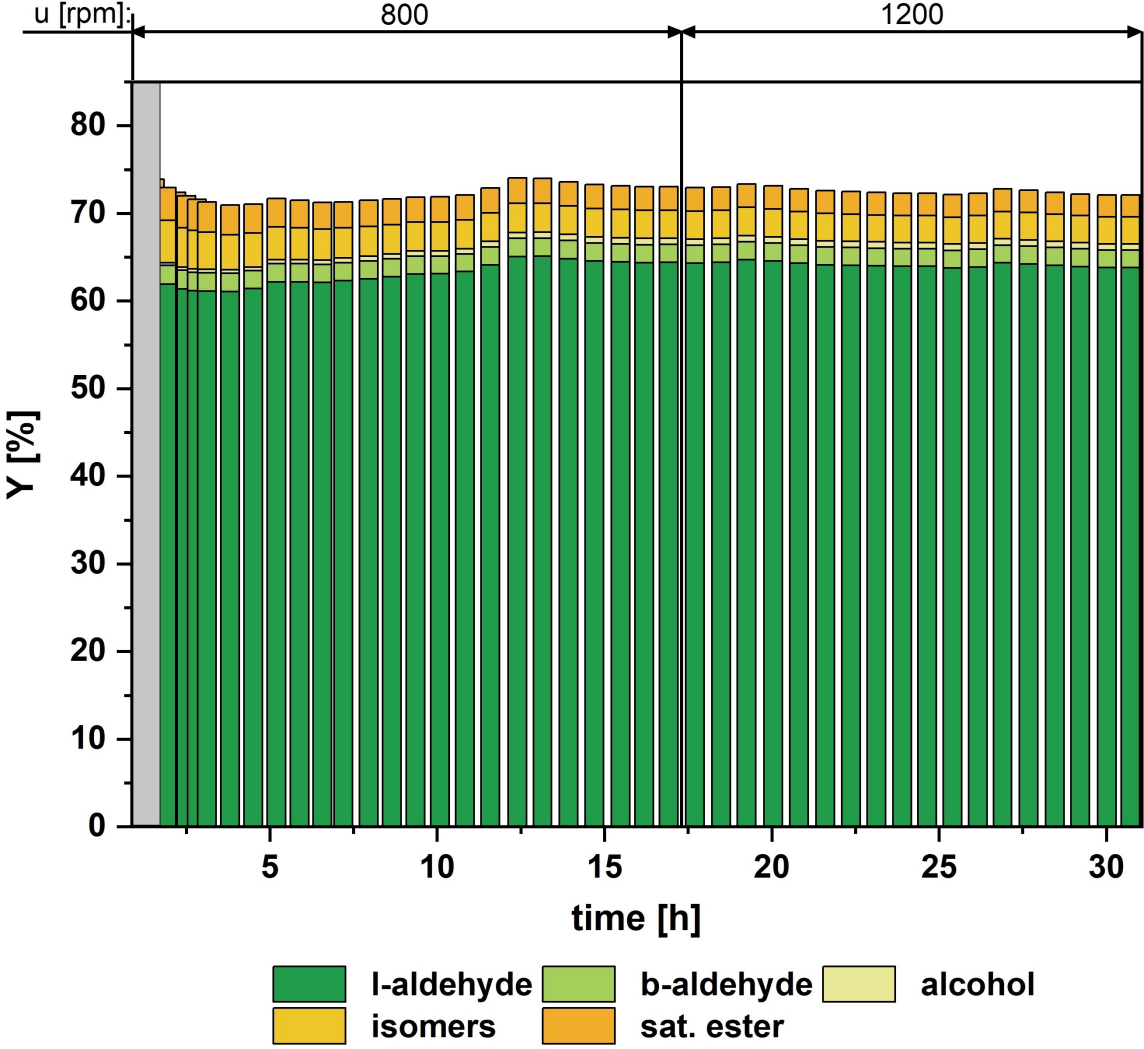
Yields of the different products in the continuous hydroformylation of M_10_U. Conversion equals the sum of all yields. Values are calculated based on GC‐FID analysis of the product stream with dibutyl ether as an internal standard. Conditions: *τ*=3 h; *T*=120 °C, *p*=30 bar; *n*_H2_/*n*_CO_=1 : 1; *u*=800–1200 rpm; *c*_Rh,aq.,0_=0.9 mmol L^−1^; *n*_1–alkene_:*n*_Rh_=2500; *n*_CD_:*n*_Rh._=665; *n*_P_:*n*_Rh_=7; *ϕ*=0.35; preforming: *T*=120 °C; *p*=15 bar; *n*_H2_/*n*_CO_=1 : 1; *u*=500 rpm; *t*>12 h.

With these preparations, all the necessary conditions for the reaction and separation have been determined to launch continuous operation (Figure 6), evaluating the achievable space‐time yield, and determine the corresponding catalyst loss. Note: Due to the kinetics in continuously stirred tank reactors compared to batch operation, full conversion cannot be reached. Hence, the residence time was set to 3 hours to achieve an efficient process with a high space‐time yield.

The continuous operation experiment was carried out with remarkable success, consistently achieving yields of up to 65 %, aldehyde selectivity of nearly 90 %, and l/b ratios of 32 for more than ten residence times. Under these optimized conditions, we produced 1.6 kg of the versatile renewable product 11‐formyl methyl undecanoate with only 63 mg of rhodium, resulting in an STY of 76.5 kg/m^3^/h ‐ a level at which the development of competitive commercial processes appears within range.^[39]^ Only 0.36 mg of rhodium was lost during this experiment, meaning >99,4 % of the initial Rh content is still in the process, while the Rh weight content in the product phase is down to 79 ppb (see SI). This corresponds to an average leaching of 0.018 % h^−1^ and the production of 4.4 kg of product per mg of catalyst lost. As in our previous work,[^35^, ^36^] no signs of leaching or degradation of CD were observed, although no detailed analytical investigation was carried out in this work. Due to the multi‐objective optimization in the batch studies aiming for a continuous process that achieves high productivity, this could be significantly increased compared to previously published processes.[^7^, ^27^, ^34^] The extreme increase in the cyclodextrin concentration allowed us to increase the phase ratio in the reactor. At the same time, the catalyst concentration was significantly reduced, doubling the yield at half of the residence time while the leaching remained at the same low level.

The successful continuous operation demonstrates that high STY can be achieved with minimal catalyst loss in these cyclodextrin‐mediated aqueous biphasic reaction systems and pave the way for sustainable hydroformylation processes. These advances underline the potential for efficient process design in producing platform chemicals from renewable raw materials. In particular, however, it also demonstrates that cyclodextrin‐mediated aqueous biphasic systems can leave their status as an academic curiosity and genuinely have the potential for competitive value‐creation in industrial processes.

## Conclusions

Intensification strategies of liquid‐liquid systems for recycling homogeneous transition metal catalysts are urgently needed and represent a major challenge in process design for the efficient continuous functionalization of renewable feedstocks such as methyl 10‐undecenoate. This publication is the culminating demonstration that cyclodextrin‐moderated aqueous biphasic systems technology is a mature and potent way to address this challenge. Using a partial factorial experimental design, we carried out extensive optimization in batch experiments. As a result, nearly quantitative conversion with >85 % selectivity was reached within 3 h. These results could also be applied directly to the hydroformylation of methyl 9‐decenoate, which opens the door to a broad downstream chemistry for both substrates. A significant achievement was that the catalyst immobilized in the aqueous phase achieved 30 % of its native activity. This system is also inherently simple to recycle, which was demonstrated in a subsequent continuous miniplant experiment in which a total STY of 76.5 kg/h/m^3^ was achieved over more than 30 hours with minimal leaching of 0.018 %/h. As a result, 4.4 kg of product could be formed per mg of catalyst lost in a pure organic phase without any contaminating organic solvents and with only down to 79 ppb of rhodium.

## Supporting Information Summary

Further information is given on the chemicals used, the detailed procedure in batch and continuous experiments, the analytics, and the implementation of the optimization. In addition, various further results on the hydroformylation of methyl 9‐decenoate, the monophasic hydroformylation of methyl 10‐undecenoate, the rheological properties of the catalyst phase at different cyclodextrin concentrations, and the leaching results in continuous experiments are presented.

## Conflict of Interests

The authors declare no conflict of interest.

1

## Supporting information

As a service to our authors and readers, this journal provides supporting information supplied by the authors. Such materials are peer reviewed and may be re‐organized for online delivery, but are not copy‐edited or typeset. Technical support issues arising from supporting information (other than missing files) should be addressed to the authors.

Supporting Information

## Data Availability

The data that support the findings of this study are available from the corresponding author upon reasonable request.
