# Supplementary material for: Continuous Production of Bifunctional Platform Chemicals From Plant Oils in Water by Cyclodextrin‐Mediated Hydroformylation
Source: ChemSusChem. 2025 Jan 23;18(10):e202402421. doi: 10.1002/cssc.202402421 (PMC12094140; doi:10.1002/cssc.202402421)
Supplement: Supplementary file 1 — Supporting Information [file CSSC-18-e202402421-s001.pdf]

ChemSusChem

Supporting Information

Continuous Production of Bifunctional Platform Chemicals From Plant Oils in Water by Cyclodextrin-Mediated Hydroformylation

Thomas Friedrich Hubertus Roth, Tobias Averbeck, Marvin Daalmann, Dieter Vogt, and Thomas Seidensticker*

Supporting Information

Continuous Production of Bifunctional Platform Chemicals from Plant Oils in Water by Cyclodextrin-Mediated Hydroformylation

Authors: Thomas Friedrich Hubertus Roth[1], Tobias Averbeck[1], Marvin Daalmann[1], D. Vogt[1] and T. Seidensticker[1*]

[1]TU Dortmund University, Department for Biochemical and Chemical Engineering, Laboratory of Industrial Chemistry, Emil-Figge-Straße 66, 44227 Dortmund, Germany

Table of Contents

1 General methods and reagents 2
1.1 Procedure for hydroformylation experiments 2
1.2 Procedure for continuous hydroformylation experiments 3
2 Analytics 3
3 Results 4
3.1 Multi-objective optimization 4
3.2 Experiment results 6
3.3 M_9D Hydroformylation 12
3.4 Monophasic $M_{10}U$ Hydroformylation 13
3.5 Properties of Catalyst Phase with varying Cyclodextrin Concentration 13
3.6 Leaching during continuous run 14

1 General methods and reagents

All chemicals used for the experiments were stored under argon, and the liquid chemicals were degassed with argon dispersed by a frit for one hour in an ultrasonic bath (Table S1).

Table S1: Chemicals used in this work.

Chemical	Manufacture	Purity [w%]
Ultrapure Water	generated with PURELAB flex 3	
Rh(acac)$(CO)_2$	Umicore AG & Co. KG	> 99.9
sulfoXantphos	MOLISA	-
Xantphos	-	-
RAME-β-Cyclodextrin	TCI	>98
Methyl-10-Undecenoate	TCI	>96
Toluol	Acros Organics	95
Dibuthylether	Acros Organics	≥ 99
Tetrahydorfuran	TCI	≥ 99.9
Syngas (1:1)	Messer SE	50 ± 2 mol% H_2, 50 ± 2 mol% CO,<br>H_2 99.999 , CO 99.997
Argon	Messer SE	99.996
N_2	Messer SE	99.5

1.1 Procedure for hydroformylation experiments

A 300 ml overhead stirred autoclave with a pitched blade stirrer, baffle and continuous gassing during the experiments was used for all batch experiments. Methyl-10-Undecenoate ($M_{10}U$) was purified on an aluminium oxide column and degassed in an ultrasonic bath by adding argon *via* a frit for 30 minutes. The same procedure was used for degassed water. Cyclodextrins were inertised by 3 cycles of vacuum application and argon pressure. The catalyst and ligand were weighed under Schlenk conditions and completely dissolved in an ultrasonic bath. Cyclodextrins are weighed under Schlenk conditions and dissolved in the ultrasonic bath at 40°C if required. Meanwhile, the reactor is inertized by 3 cycles of vacuuming and pressurisation with 10 bar of nitrogen pressure. The catalyst solution is transferred into the reactor via vacuum, and preforming is started. The substrate is transferred in counterflow into a substrate reservoir attached above the reactor, pressurised with synthesis gas and fed into the reactor at the end of preforming by opening the connective valve. At the end of the experiment, the gas supply is closed, the stirrer speed is reduced to 400 rpm, and the reactor is cooled with ice water. The reactor is then degassed and flushed several times with argon, the phases are separated, and the corresponding analyses are carried out. Finally, the reactor and periphery are cleaned with isopropanol and stored in a drying oven.

1.2 Procedure for continuous hydroformylation experiments

The miniplant used has already been described in detail in our previous publication and is briefly described here for convenience.

The Miniplant consists of a 2100 ml reactor and a decanter. The reactor is equipped with a magnetically coupled overhead stirrer, a baffle and a double-walled heating jacket. Various gases (CO, H_2, Ar, N_2) can be dosed *via* mass flow controllers, and liquids can be dosed *via* HPLC pumps. A pipe installed at the height of the stirrer pumps the reaction solution into the decanter, which is also tempered by a double-walled jacket. Here, the mixture is separated into a pure organic phase (top), which leaves the system via a flash, and the aqueous catalyst phase (bottom), which flows back into the reactor gravimetrically.

The substrate and catalyst solution are prepared and weighed as in the batch experiments. The miniplant is inertized by repeatedly applying a vacuum and 5 bar nitrogen pressure. The entire catalyst solution is drawn into the reactor *via* vacuum, the entire miniplant is pressurised with 15 bar syngas, and the preforming is carried out at 120 °C and 500 rpm overnight.

The calculated amount of catalyst solution is pumped into the decanter, and the latter is tempered to the separation temperature. The substrate is pumped into the reactor at the highest possible pump capacity until the desired phase ratio is reached. After 3 hours of batch operation, the system is switched to continuous operation.

2 Analytics

Experimental samples were analysed on an Agilent Technologies gas chromatograph (7890A) equipped with a flame ionisation detector and an HP-5 capillary column (30 m x 0.32 mm x 0.25 µm, methods in Table S2 and Table S3). Tetrahydrofuran (THF) 0.6 g solvent, dibutyl ether 0.05 g internal standard and 0.35 g sample were used for the analysis.

Table S2: Heating profiles for the analysis of hydroformylation using GC-FID with HP-5 column.

	**Rate [°C min^{-1}]**	**Temperature [°C]**	**Holding time [min]**
T_{Start}		40	2
**Ramp 1**	50	100	1
**Ramp 2**	10	200	2
**Ramp 3**	50	320	2

Table S3: Volume flows via a column for the analysis of hydroformylation using GC-FID with HP-5 column.

	**Rate [ml min^{-2}]**	**Value [ml min^{-1}]**	**Holding time [min]**
n_{Start}		2.19	5
**Ramp 1**	1	1	5
**Ramp 2**	1	2.5	0

The loss of rhodium and phosphorus in the organic phase was determined by Analytic Jena's inductively coupled plasma optical emission spectrometer (Plasma Quant PQ 9000). Before analysis, the samples were concentrated *in vacuo* and at elevated temperature to approx. 1/10 of the original volume.

3 Results

3.1 Multi-objective optimization

An equation is developed in which the four individual terms (leaching (j=1), selectivity (j=2), RZA (j=3), TON (j=4)) are standardized with the overall highest $x_{j,max}$ and lowest $x_{j,min}$ reached values to form a target variable $Z_{1,i}$ (equation 1). The values of the respective influencing variable $x_{j,i}$ contained in the terms are also assigned weighting factors w_j. Leaching is optimized by introducing an exponential function so that this term has a high influence on the target function even with a small increase.

$$Z_{1,i} = w_1 \cdot \left|\frac{x_{1,i} - x_{1,min}}{x_{1,max} - x_{1,min}}\right| + w_2 \cdot \left|\frac{x_{2,i} - x_{2,min}}{x_{2,max} - x_{2,min}}\right| + w_3 \cdot \left|\frac{x_{3,i} - x_{3,min}}{x_{3,max} - x_{3,min}}\right| + w_4 \cdot \left|\frac{x_{4,i} - x_{4,min}}{x_{4,max} - x_{4,min}}\right| \quad (1)$$

In order to achieve a balanced fulfillment of all target variables, a penalty term $Z_{2,i}$ (equation 2) is also introduced.

$$Z_{2,i} = \frac{1}{\ln\left(\frac{w_1}{x_{1,i} + 10^{-7}} + \frac{w_2}{x_{2,i} + 10^{-7}} + \frac{w_3}{x_{3,i} + 10^{-7}} + \frac{w_4}{x_{4,i} + 10^{-7}}\right)} \quad (2)$$

The target values determined for both equations are then added together, normalized in each case and combined to form an overall target function $Z_{tot,i}$ (equation 3).

$$Z_{tot.,i} = \frac{1}{2} \cdot \left(\frac{Z_{1,i} - Z_{1,min}}{Z_{1,max} - Z_{1,min}} + \frac{Z_{2,i} - Z_{2,min}}{Z_{2,max} - Z_{2,min}}\right) \quad (3)$$

Based on this calculation, each sample in every experiment is assigned an overall target value. The leaching value of the overall experiment was assigned to every sample within the given experiment. The overall target value $Z_{tot.,i}$ is then used in the next step to fit a model on these data using Gaussian process regression.

The model obtained here is then forwarded to the two-stage optimization approach:

```
% Optimized Chemical Parameters
% This script performs a two-stage optimization (Genetic Algorithm + Global
Search)
% to find optimal parameters for a chemical process.

% Load data and model
% Replace these lines with your actual data loading method
data = load('your_data_file.mat');  % Load your data
X = data.X;  % Input variables
Y = data.Y;  % Output variable
load('your_trained_model.mat');  % Load your trained model

% Define test points for each parameter
% Adjust these based on your specific parameter ranges
k1_Test = [20 35 50]';
k2_Test = [2 10 50]';
k3_Test = [2 10 20]';
k4_Test = [150 420 700]';
k5_Test = [0.0006 0.0014 0.0022]';

% Create full factorial design of experiments
```

```
dFF = fullfact([length(k1_Test) length(k2_Test) length(k3_Test)
length(k4_Test) length(k5_Test)]);

% Assign actual values to the factorial design
param_values = {k1_Test, k2_Test, k3_Test, k4_Test, k5_Test};
for i = 1:size(dFF, 2)
    dFF(:,i) = param_values{i}(dFF(:,i));
end

% Define fitness function
% Note: We negate the output because we want to maximize, but optimization
functions minimize by default
fitnessFunction = @(x) -chemicalTest(x, trainedModel);

% Set up genetic algorithm options
numberOfVariables = size(X, 2);
options = optimoptions('ga', 'MaxGenerations', 1000, 'Display', 'iter');

% Define bounds based on your data
lb = min(X);  % Lower bound
ub = max(X);  % Upper bound

% Run genetic algorithm
[x_opt_GA, fval] = ga(fitnessFunction, numberOfVariables, [], [], [], [],
lb, ub, [], [], options);

% Perform global search for each starting point in the factorial design
results = zeros(size(dFF, 1), 13);
for b = 1:size(dFF, 1)
    fprintf('Run: %d of %d\n', b, size(dFF,1))

    % Set starting point from factorial design
    x0 = dFF(b, 1:5);

    % Set up optimization problem
    problem = createOptimProblem('fmincon', 'objective', fitnessFunction,
...
        'x0', x0, 'lb', lb, 'ub', ub);

    % Perform global search
    gs = GlobalSearch;
    [xg, fg, exitflag, output, ~] = run(gs, problem);

    % Store results
    results(b, 1:5) = dFF(b, 1:5);  % Starting points
    results(b, 6) = -100*fg;  % Objective function value (negated and
scaled)
    results(b, 7:11) = xg;  % Optimal parameters
    results(b, 12) = output.localSolverSuccess;  % Number of successful
local solvers
    results(b, 13) = output.localSolverTotal;  % Total number of local
solvers
end

% Define the chemicalTest function using the trained model
function y = chemicalTest(x, Mdl)
    y = predict(Mdl, x);
end
```

3.2 Experimental results

Experiment	time [min]	T [°C]	n_{CD}:n_{Rh}	c_{Rh} [mmol L^{-1}]	ϕ	X	$Y_{l\text{-}aldehyd}$
10	5	120	98,8	2,245	0,2	12,8	10,5
	15	120	98,8	2,245	0,2	33,0	28,2
	30	120	98,8	2,245	0,2	57,1	49,1
	60	120	98,8	2,245	0,2	79,6	68,9
	120	120	98,8	2,245	0,2	95,6	83,0
	180	120	98,8	2,245	0,2	99,1	85,8
	240	120	98,8	2,245	0,2	100,0	86,0
1	5	100	100,0	0,561	0,2	3,0	1,2
	15	100	100,0	0,561	0,2	1,6	0,6
	30	100	100,0	0,561	0,2	3,4	2,6
	60	100	100,0	0,561	0,2	5,8	4,9
	120	100	100,0	0,561	0,2	11,2	9,3
	180	100	100,0	0,561	0,2	16,1	13,6
	240	100	100,0	0,561	0,2	20,9	18,2
21	5	140	500,1	0,561	0,2	19,5	16,1
	15	140	500,1	0,561	0,2	50,4	42,6
	30	140	500,1	0,561	0,2	79,7	68,0
	60	140	500,1	0,561	0,2	95,8	82,1
	120	140	500,1	0,561	0,2	99,4	84,3
	180	140	500,1	0,561	0,2	100,0	83,5
	240	140	500,1	0,561	0,2	100,0	82,3
28	5	160	250,0	0,561	0,2	49,9	38,7
	15	160	250,0	0,561	0,2	81,9	65,1
	30	160	250,0	0,561	0,2	96,3	76,6
	60	160	250,0	0,561	0,2	99,4	77,2
	120	160	250,0	0,561	0,2	100,0	75,0
	180	160	250,0	0,561	0,2	100,0	70,6
	240	160	250,0	0,561	0,2	100,0	67,5
12	5	120	249,3	0,563	0,4	4,0	3,0
	15	120	249,3	0,563	0,4	5,4	4,4
	30	120	249,3	0,563	0,4	10,3	8,7
	60	120	249,3	0,563	0,4	20,1	16,9
	120	120	249,3	0,563	0,4	34,5	30,0
	180	120	249,3	0,563	0,4	45,9	39,4
	240	120	249,3	0,563	0,4	55,4	47,0
22	5	140	99,7	1,126	0,4	10,6	8,9
	15	140	99,7	1,126	0,4	29,3	24,7
	30	140	99,7	1,126	0,4	47,9	41,1
	60	140	99,7	1,126	0,4	70,9	60,7
	120	140	99,7	1,126	0,4	90,5	77,1
	180	140	99,7	1,126	0,4	96,6	81,4
	240	140	99,7	1,126	0,4	98,7	82,6
24	5	140	99,7	1,126	0,6	6,4	5,0

	15	140	99,7	1,126	0,6	16,1	12,7
	30	140	99,7	1,126	0,6	29,1	23,4
	60	140	99,7	1,126	0,6	48,5	38,5
	120	140	99,7	1,126	0,6	71,9	55,2
	180	140	99,7	1,126	0,6	85,0	63,0
	240	140	99,7	1,126	0,6	95,3	67,7
5	5	100	250,4	2,243	0,6	1,9	1,2
	15	100	250,4	2,243	0,6	3,3	2,8
	30	100	250,4	2,243	0,6	6,1	5,1
	60	100	250,4	2,243	0,6	11,3	9,7
	120	100	250,4	2,243	0,6	20,3	17,8
	180	100	250,4	2,243	0,6	28,5	24,4
	240	100	250,4	2,243	0,6	35,6	30,5
19	5	140	249,9	2,247	0,2	74,6	39,1
	15	140	249,9	2,247	0,2	96,0	56,0
	30	140	249,9	2,247	0,2	99,0	58,1
	60	140	249,9	2,247	0,2	100,0	58,9
	120	140	249,9	2,247	0,2	100,0	58,1
	180	140	249,9	2,247	0,2	100,0	52,8
	240	140	249,9	2,247	0,2	100,0	54,3
3	5	100	100,0	2,246	0,4	2,7	1,9
	15	100	100,0	2,246	0,4	3,5	3,0
	30	100	100,0	2,246	0,4	6,5	5,5
	60	100	100,0	2,246	0,4	12,7	10,9
	120	100	100,0	2,246	0,4	23,7	20,2
	180	100	100,0	2,246	0,4	31,3	27,4
	240	100	100,0	2,246	0,4	39,2	34,3
14	5	120	249,3	1,126	0,6	2,7	2,2
	15	120	249,3	1,126	0,6	8,2	6,2
	30	120	249,3	1,126	0,6	17,6	12,3
	60	120	249,3	1,126	0,6	30,1	21,1
	120	120	249,3	1,126	0,6	52,4	34,9
	180	120	249,3	1,126	0,6	67,7	43,7
	240	120	249,3	1,126	0,6	78,7	49,7
25	5	140	747,9	0,563	0,6	8,9	4,7
	15	140	747,9	0,563	0,6	25,0	11,8
	30	140	747,9	0,563	0,6	45,5	19,9
	60	140	747,9	0,563	0,6	70,2	28,8
	120	140	747,9	0,563	0,6	91,4	36,9
	180	140	747,9	0,563	0,6	95,9	37,7
	240	140	747,9	0,563	0,6	96,7	39,3
31	5	160	249,3	0,563	0,4	19,6	15,4
	15	160	249,3	0,563	0,4	44,9	36,2
	30	160	249,3	0,563	0,4	68,2	54,6
	60	160	249,3	0,563	0,4	87,5	69,1
	120	160	249,3	0,563	0,4	96,8	70,6

	180	160	249,3	0,563	0,4	97,8	70,3
	240	160	249,3	0,563	0,4	97,8	69,5
20	15	140	249,9	2,247	0,2	91,1	61,5
	30	140	249,9	2,247	0,2	98,8	67,1
	60	140	249,9	2,247	0,2	100,0	69,4
	120	140	249,9	2,247	0,2	100,0	66,8
	180	140	249,9	2,247	0,2	100,0	64,2
	240	140	249,9	2,247	0,2	100,0	62,3
15	5	120	500,8	2,243	0,6	7,9	6,4
	15	120	500,8	2,243	0,6	22,8	18,9
	30	120	500,8	2,243	0,6	38,4	33,0
	60	120	500,8	2,243	0,6	58,4	50,1
	120	120	500,8	2,243	0,6	81,8	69,9
	180	120	500,8	2,243	0,6	92,2	78,5
	240	120	500,8	2,243	0,6	96,6	82,0
13	5	120	747,9	1,126	0,4	9,6	7,4
	15	120	747,9	1,126	0,4	23,0	19,3
	30	120	747,9	1,126	0,4	45,9	39,0
	60	120	747,9	1,126	0,4	68,3	58,0
	120	120	747,9	1,126	0,4	90,3	76,9
	180	120	747,9	1,126	0,4	96,6	82,1
	240	120	747,9	1,126	0,4	98,7	83,5
34	5	160	498,6	1,126	0,6	28,9	20,0
	15	160	498,6	1,126	0,6	76,1	53,1
	30	160	498,6	1,126	0,6	92,5	58,7
	60	160	498,6	1,126	0,6	96,6	59,4
	120	160	498,6	1,126	0,6	97,7	62,5
	180	160	498,6	1,126	0,6	97,3	62,1
	240	160	498,6	1,126	0,6	98,3	63,0
4	5	100	498,6	0,563	0,4	1,7	1,2
	15	100	498,6	0,563	0,4	3,6	3,1
	30	100	498,6	0,563	0,4	7,0	5,7
	60	100	498,6	0,563	0,4	12,5	10,6
	120	100	498,6	0,563	0,4	22,7	18,8
	180	100	498,6	0,563	0,4	32,6	28,1
	240	100	498,6	0,563	0,4	38,5	33,3
8	5	100	498,6	1,126	0,8	2,0	0,8
	15	100	498,6	1,126	0,8	3,3	1,3
	30	100	498,6	1,126	0,8	5,4	2,2
	60	100	498,6	1,126	0,8	9,0	3,7
	120	100	498,6	1,126	0,8	15,8	6,6
	180	100	498,6	1,126	0,8	21,8	9,2
	240	100	498,6	1,126	0,8	27,4	11,9
9	5	100	747,9	2,252	0,8	1,6	1,1
	15	100	747,9	2,252	0,8	2,8	2,0
	30	100	747,9	2,252	0,8	5,0	4,0

	60	100	747,9	2,252	0,8	8,3	6,6
	120	100	747,9	2,252	0,8	15,9	12,2
	180	100	747,9	2,252	0,8	22,5	18,0
	240	100	747,9	2,252	0,8	28,5	22,6
23	5	140	500,1	2,246	0,4	59,3	25,2
	15	140	500,1	2,246	0,4	91,7	47,9
	30	140	500,1	2,246	0,4	98,8	53,8
	60	140	500,1	2,246	0,4	100,0	54,1
	120	140	500,1	2,246	0,4	100,0	53,6
	180	140	500,1	2,246	0,4	100,0	53,2
	240	140	500,1	2,246	0,4	100,0	52,2
7	5	100	249,3	1,126	0,8	1,9	0,9
	15	100	249,3	1,126	0,8	2,7	0,9
	30	100	249,3	1,126	0,8	4,4	1,5
	60	100	249,3	1,126	0,8	8,3	3,0
	120	100	249,3	1,126	0,8	13,6	5,0
	180	100	249,3	1,126	0,8	19,6	7,2
	240	100	249,3	1,126	0,8	24,6	9,2
2	5	100	500,8	1,121	0,2	2,8	2,1
	15	100	500,8	1,121	0,2	9,6	7,1
	30	100	500,8	1,121	0,2	22,3	17,8
	60	100	500,8	1,121	0,2	42,0	33,7
	120	100	500,8	1,121	0,2	67,7	55,8
	180	100	500,8	1,121	0,2	81,0	67,4
	240	100	500,8	1,121	0,2	91,0	75,8
36	5	160	498,6	2,252	0,8	26,0	15,7
	15	160	498,6	2,252	0,8	59,4	32,6
	30	160	498,6	2,252	0,8	80,4	39,6
	60	160	498,6	2,252	0,8	94,6	45,0
	120	160	498,6	2,252	0,8	96,2	46,0
	180	160	498,6	2,252	0,8	95,4	45,8
	240	160	498,6	2,252	0,8	94,5	45,8
27	5	140	747,9	0,563	0,8	8,1	1,9
	15	140	747,9	0,563	0,8	17,4	2,6
	30	140	747,9	0,563	0,8	31,1	3,6
	60	140	747,9	0,563	0,8	51,1	5,2
	120	140	747,9	0,563	0,8	72,5	7,2
	180	140	747,9	0,563	0,8	91,0	8,8
	240	140	747,9	0,563	0,8	92,4	8,9
26	5	140	249,3	1,126	0,8	7,6	2,5
	15	140	249,3	1,126	0,8	17,3	4,4
	30	140	249,3	1,126	0,8	32,4	6,7
	60	140	249,3	1,126	0,8	53,2	9,5
	120	140	249,3	1,126	0,8	73,4	11,7
	180	140	249,3	1,126	0,8	88,1	13,3
	240	140	249,3	1,126	0,8	93,7	13,8

30	5	160	99,7	1,126	0,4	39,7	31,2
	15	160	99,7	1,126	0,4	72,9	58,3
	30	160	99,7	1,126	0,4	91,9	73,9
	60	160	99,7	1,126	0,4	98,9	78,4
	120	160	99,7	1,126	0,4	100,0	77,7
	180	160	99,7	1,126	0,4	100,0	75,7
	240	160	99,7	1,126	0,4	100,0	74,4
18	5	120	498,6	0,563	0,8	3,7	1,2
	15	120	498,6	0,563	0,8	7,1	1,7
	30	120	498,6	0,563	0,8	11,6	2,4
	60	120	498,6	0,563	0,8	23,0	4,4
	120	120	498,6	0,563	0,8	36,5	6,6
	180	120	498,6	0,563	0,8	50,4	9,0
	240	120	498,6	0,563	0,8	60,0	10,4
11	5	120	751,2	1,121	0,2	20,0	13,6
	15	120	751,2	1,121	0,2	50,5	38,7
	30	120	751,2	1,121	0,2	78,5	62,2
	60	120	751,2	1,121	0,2	94,7	75,5
	120	120	751,2	1,121	0,2	100,0	78,4
	240	120	751,2	1,121	0,2	100,0	77,2
29	5	160	750,2	1,121	0,2	85,8	37,8
	15	160	750,2	1,121	0,2	100,0	52,1
	30	160	750,2	1,121	0,2	100,0	53,7
	60	160	750,2	1,121	0,2	100,0	53,5
	120	160	750,2	1,121	0,2	100,0	50,8
	180	160	750,2	1,121	0,2	100,0	47,9
	240	160	750,2	1,121	0,2	100,0	43,6
16	5	120	751,2	2,243	0,6	10,9	8,5
	15	120	751,2	2,243	0,6	24,2	20,4
	30	120	751,2	2,243	0,6	40,9	34,5
	60	120	751,2	2,243	0,6	66,0	55,7
	120	120	751,2	2,243	0,6	86,5	73,4
	180	120	751,2	2,243	0,6	94,8	80,2
	240	120	751,2	2,243	0,6	97,9	82,6
6	5	100	747,9	0,563	0,6	1,8	0,8
	15	100	747,9	0,563	0,6	2,8	1,7
	30	100	747,9	0,563	0,6	4,9	3,1
	60	100	747,9	0,563	0,6	9,2	5,7
	120	100	747,9	0,563	0,6	16,7	10,9
	180	100	747,9	0,563	0,6	23,2	15,0
	240	100	747,9	0,563	0,6	29,6	19,0
35	5	160	99,7	2,252	0,8	11,0	7,8
	15	160	99,7	2,252	0,8	25,9	20,2
	30	160	99,7	2,252	0,8	44,0	33,8
	60	160	99,7	2,252	0,8	70,0	42,5
	120	160	99,7	2,252	0,8	92,3	47,0

	180	160	99,7	2,252	0,8	96,3	48,1
	240	160	99,7	2,252	0,8	95,6	48,2
17	5	120	99,7	0,563	0,8	2,2	0,4
	15	120	99,7	0,563	0,8	4,8	0,6
	30	120	99,7	0,563	0,8	8,8	0,9
	60	120	99,7	0,563	0,8	16,9	1,5
	120	120	99,7	0,563	0,8	31,7	2,7
	180	120	99,7	0,563	0,8	40,8	3,4
	240	120	99,7	0,563	0,8	50,5	4,3
32	5	160	750,1	2,246	0,4	48,1	11,6
	15	160	750,1	2,246	0,4	76,7	23,0
	30	160	750,1	2,246	0,4	100,0	34,9
	60	160	750,1	2,246	0,4	100,0	35,6
	120	160	750,1	2,246	0,4	100,0	38,8
	180	160	750,1	2,246	0,4	100,0	39,8
	240	160	750,1	2,246	0,4	100,0	39,8
Optimum	5	120	663,1	2,150	0,5	11,2	9,2
	15	120	663,1	2,150	0,5	27,7	23,5
	30	120	663,1	2,150	0,5	46,7	39,9
	45	120	663,1	2,150	0,5	60,6	51,9
	60	120	663,1	2,150	0,5	70,4	60,4
	90	120	663,1	2,150	0,5	85,3	73,2
	120	120	663,1	2,150	0,5	90,9	78,0
	150	120	663,1	2,150	0,5	94,8	81,4
	180	120	663,1	2,150	0,5	97,1	83,4
	210	120	663,1	2,150	0,5	98,4	84,4
	240	120	663,1	2,150	0,5	99,0	84,7
Conditions Künnemann	5	120	13,1	2,247	0,2	3,1	3,1
	15	120	13,1	2,247	0,2	8,9	8,9
	30	120	13,1	2,247	0,2	17,5	17,5
	60	120	13,1	2,247	0,2	32,0	29,9
	90	120	13,1	2,247	0,2	42,3	38,8
	120	120	13,1	2,247	0,2	52,3	48,2
	150	120	13,1	2,247	0,2	62,5	58,8
	180	120	13,1	2,247	0,2	66,2	61,1
	210	120	13,1	2,247	0,2	71,9	66,4
	240	120	13,1	2,247	0,2	76,3	70,5

3.3 M9D Hydroformylation

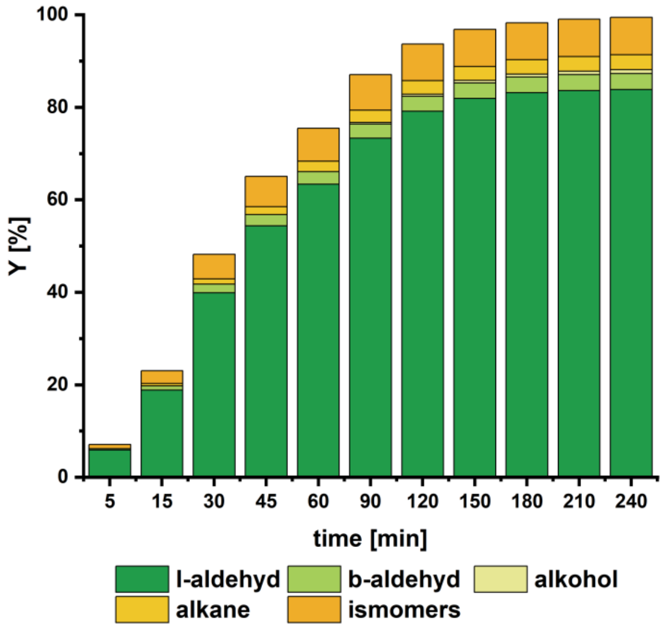

Figure S 1: Yield over time for the hydroformylation of M9D under the optimized conditions for the hydroformylation of M10U. Conversion equals the sum of all yields. Yields are calculated based on GC-FID analysis of the product mixture with dibutyl ether as an internal standard. Conditions: T = 120 °C, p = 30 bar, u = 800 min^{-1}, n_{CO}:n_{H2} = 1:1, $c_{Rh,aq.}$= 0.9 mmol L^{-1}, n_{CD}:n_{Rh} = 665, φ = 0.34, n_{Sub}:n_{Rh} = 2500, n_P:n_{Rh} = 7; Preforming: T = 120 °C, p = 20 bar, u = 800 min^{-1}, t = 1 h;

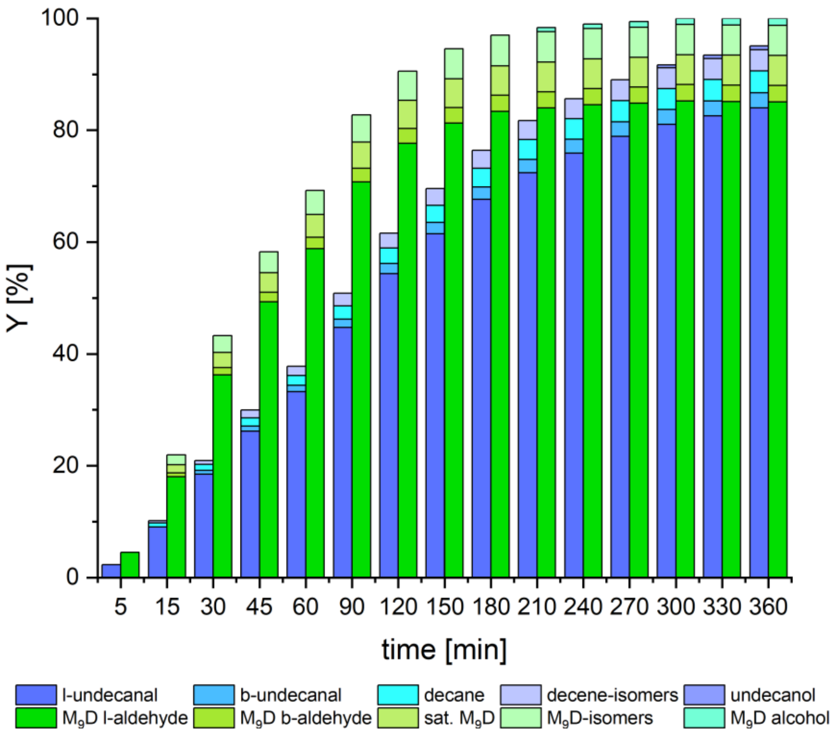

Figure S 2: Yield over time for the hydroformylation of an equimolar mixture of M9D and 1-Decene under the optimized conditions for the hydroformylation of M10U. Conversion equals the sum of all yields. Yields are calculated based on GC-FID analysis of the product mixture with dibutyl ether as an internal standard. Conditions: T = 120 °C, p = 30 bar, u = 800 min^{-1}, n_{CO}:n_{H2} = 1:1, $c_{Rh,aq.}$= 0.9 mmol L^{-1}, n_{CD}:n_{Rh} = 665, φ = 0.34, n_{Sub}:n_{Rh} = 2500, n_P:n_{Rh} = 7; Preforming: T = 120 °C, p = 20 bar, u = 800 min^{-1}, t = 1 h;

3.4 Monophasic $M_{10}U$ Hydroformylation

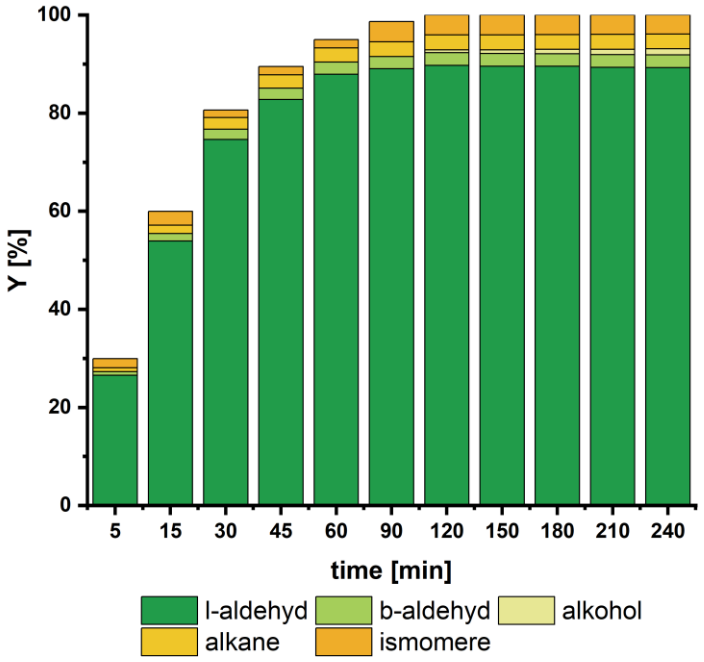

Figure S 3: Yield over time for the hydroformylation of $M_{10}U$ in toluene with Xantphos as ligand. Conversion equals the sum of all yields. Yields are calculated based on GC-FID analysis of the product mixture with dibutyl ether as an internal standard. Conditions: T = 120 °C, p = 30 bar, u = 800 min^{-1}, n_{CO}:n_{H2} = 1:1, c_{Rh} = 1.0 mmol L^{-1}, n_{CD}:n_{Rh} = 0, n_{Sub}:n_{Rh} = 2500, n_P:n_{Rh} = 7; Preforming: T = 120 °C, p = 20 bar, u = 800 min^{-1}, t = 1 h;

3.5 Properties of Catalyst Phase with varying Cyclodextrin Concentration

The densities were measured by Ms. Susanne Richter using a DMA 4100M density meter from Anton Paar at the Chair of Thermodynamics at the Technical University of Dortmund. Measurements above 90°C form bubbles that affect the oscillation for the density measurement, which is therefore not possible.

The viscosities were measured using a Lovis 2000 M/ME ball-rolling viscometer from Anton Paar by Ms. Susanne Richter at the Chair of Thermodynamics at the TU Dortmund.

Table S4: Density of aqueous cyclodextrin solutions.

m_{CD}/m_{H2O} [-]	0.1	0.25	0.5	1	1.5	1.88	2	2.5
T [°C]	[g/cm³]	[g/cm³]	[g/cm³]	[g/cm³]	[g/cm³]	[g/cm³]	[g/cm³]	[g/cm³]
13	1.0257	1.0598	1.1038	1.1631	1.1999	1.2295	1.2263	1.2426
20	1.0242	1.0579	1.1012	1.1596	1.1958	1.225	1.2219	1.238
30	1.0213	1.0544	1.0969	1.1541	1.1896	1.2183	1.2153	1.2311
40	1.0175	1.0501	1.092	1.1483	1.1832	1.2114	1.2084	1.2241
50	1.013	1.0452	1.0865	1.142	1.1764	1.2043	1.2013	1.2168
60	1.0075	1.0399	1.0806	1.1354	1.1693	1.197	1.194	1.2093
70	1.0021	1.0335	1.0738	1.1283	1.1615	1.1893	1.1864	1.2016
80	0.9952	1.0268		1.1206	1.1545	1.1815	1.1786	1.1937

Table S5: Dynamic viscosities of aqueous cyclodextrin solutions.

m_{CD}/m_{H2O} [-]	0.1	0.25	0.5	1	1.5	1.88	2	2.5
	[mPa·s]	[mPa·s]	[mPa·s]	[mPa·s]	[mPa·s]	[mPa·s]	[mPa·s]	[mPa·s]
13	1.6696	2.8718	7.2429	53.729	367.73	44042	25856	
20	1.3798	2.3102	5.5637	36.757	219.99	21379	13246	
30	1.0834	1.7597	4.0112	22.938	116	8865	5807.2	22479
40	0.88043	1.389	3.0236	15.248	67.4	4440.5	2912	9678.8
50	0.73297	1.1291	2.3606	10.642	41.958	2291.7	1591.5	4621.9
60	0.62672	0.94193	1.896	8.1393	27.671	1301.3	940.71	2475.6
70	0.54363	0.80227	1.5581	6.0196	19.131	784.02	592.3	1428.6
80	0.47804	0.692	1.3082	4.6093	13.71	496.48	392.1	879.27

3.6 Leaching during continuous run

Table S6: Leaching results in the continuously operated hydroformylation of $M_{10}U$.

time [h]		Rhodium			Phosphorus		
from	to	w_{org} [ppb]	m [mg/h]	L [%/h]	w_{org} [ppb]	m [mg/h]	L [%/h]
0	2.2	379	0.027	0.0427	336	0.024	0.0180
2.3	4.8	176	0.013	0.0204	145	0.011	0.0080
4.9	7.6	212	0.015	0.0244	223	0.016	0.0122
7.7	10.5	121	0.009	0.0136	161	0.011	0.0086
10.6	12.8	122	0.008	0.0132	117	0.008	0.0060
12.9	15.1	149	0.010	0.0158	159	0.011	0.0080
15.2	17.7	79	0.005	0.0085	77	0.005	0.0039
17.8	20.1	89	0.006	0.0093	84	0.006	0.0042
20.2	22.4	145	0.010	0.0153	146	0.010	0.0073
22.5	24.7	175	0.012	0.0191	154	0.011	0.0080
24.8	26.9	263	0.018	0.0285	257	0.018	0.0132
27.0	29.6	85	0.006	0.0091	82	0.006	0.0041
29.7	32.2	118	0.009	0.0137	53	0.004	0.0029
